# Anti-Lipase Potential of the Organic and Aqueous Extracts of Ten Traditional Edible and Medicinal Plants in Palestine; a Comparison Study with Orlistat

**DOI:** 10.3390/medicines4040089

**Published:** 2017-12-08

**Authors:** Nidal Jaradat, Abdel Naser Zaid, Fatima Hussein, Maram Zaqzouq, Hadeel Aljammal, Ola Ayesh

**Affiliations:** Department of Pharmacy, Faculty of Medicine and Health Sciences, An-Najah National University, P.O. Box 7, Nablus 00970, Palestine; anzaid@najah.edu (A.N.Z.); f.huseen@najah.edu (F.H.); maram-zaqzouq@hotmail.com (M.Z.); hadeeljawad133@outlook.com (H.A.); oayesh@najah.edu (O.A.)

**Keywords:** anti-obesity, anti-lipase, traditional medicine, folkloric food

## Abstract

**Background:** Herbs have played a fundamental and essential role in the humans life since ancient times, especially those which are used as food and/or folk medicinedue to both their nutritive and curative properties.This study aimed to investigate new antilipase agents from tentraditional Palestinian edible and medicinal plants through inhibition of the absorption of dietary lipids. **Methods:** The anti-lipase activity for ten plants was evaluated and compared with the reference compound Orlistat by using the porcine pancreatic lipase inhibitory test which was conducted by using a UV-visible spectrophotometer. **Results:** The aqueous extracts of *Vitis vinifera* and *Rhus coriaria* had the highest antilipase effects with IC_50_ values 14.13 and 19.95 mcg/mL, respectively. Meanwhile, the organic extract of *Origanum dayi* had an IC_50_ value 18.62 mcg/mL. *V. vinifera* showed the highest porcine pancreatic lipase inhibitory effects when compared with Orlistat, which has an IC_50_ value 12.38 mcg/mL. **Conclusions:** According to the obtained results, *V. vinifera*, *R. coriaria*, and *O. dayi* can be considered a natural inhibitors of the pancreatic lipase enzyme as well as new players in obesity treatment. In fact, these plants can be freely and safely consumed in a daily diet or can be prepared as nutraceutical formulations to treat or prevent of obesity.

## 1. Introduction

The results from the WHO global survey on traditional, complementary/alternative, and herbal medicines showed that the market for these kinds of medicines is growing steadily worldwide. In fact, the usage of phytopharmaceuticals and nutraceuticals is rapidly and continuously expanding. Recently, many people have been using these formulations in the treatment or prevention of various diseases and disorders in different national healthcare systems. Moreover, many patients often use herbal medicines to complement treatment with conventional medicines [1,2,3]. Herbals and phytopharmaceuticals for the treatment of excess weightand obesity were among the most used remedies, especially in developing and developed countries, since these metabolic disorders became very prominent [4,5]. In fact, obesity poses a worldwide concern, not only for the harm which excess weight alone may cause, but also due to associated health problems such as endocrine, metabolic, and cardiovascular disorders [6,7,8].

Accordingly, various therapeutic protocols are utilized globally to control excess body weight and hyperlipidemia in obese patients. In fact, many drugs which have recently become available in pharmaceutical markets are recommended for use hand-in-hand with diet and exercise changes for the reduction of body weight and decreasing lipid levels in the plasma [6,9].

Orlistat is prototype weight loss drug. It is a gastrointestinal lipase inhibitor that competes with dietary fats for sites on the lipase molecules and has been shown to block the absorption of around 30% of dietary fat at a therapeutic oral dose of 120 mg, three times a day. Orlistat inhibits the hydrolysis of dietary triglycerides and thus reduces the subsequent intestinal absorption of the products of lipolysis (monoglycerides and free fatty acids), and does not demonstrate any efficient effect on appetite [10].Therefore, one of the most important screening strategies in the discovery of anti-obesity formulations is to search for potent lipase inhibitors from natural plant extracts [10].

Traditional medicinal plants have been used for obesity and body weight control in many countries. In fact, their consumption, along with appropriate dietary changes, is becoming one of the most popular complementary and alternative medicine strategies for the control of obesity and weight gain [11,12].

In this study, the organic and the aqueous extracts of various edible and traditional medicinal plants, which were collected from different regions of Palestine, were screened as potential anti-obesity agents by monitoring their anti-lipase activity. A total of 10 plants belonging to nine families from Palestine, which are used both as folk medicine and food, were studied, including: *Arum palaestinum* Boiss., *Crataegus azarolus* L., *Malva parviflora* L., *Taraxacum syriacum* Boiss., *Rhus coriaria* L., *Rosmarinus officinalis* L., *Psidium guajava* L., *Origanum dayi* Post, *Brassica nigra* (L.) K. Koch, and *Vitis vinifera* L. The first nine plants grow wildly in the mountains of Palestine and most of them are used in folk medicine to control weight gain [13,14].

In this study, the tested plants were evaluated for their antilipase activity by using a simple, fast, efficient, and reliable spectrophotometric method, in an attempt to investigate these new agents for their ability to impair the of digestion and assimilation of dietary fats. In addition, they were compared with Orlistat in order to assess their potential use as an alternative to this chemical agent.

## 2. Materials and Methods

### 2.1. Instrumentation

Shaker device (Memmert shaking incubator, Buchenbach, Germany), UV-visible spectrophotometer (Jenway 7135, Staffordshire, UK), grinder (Moulinex, model LM2211, UNO, Shanghai, China), balance (Rad wag, AS 220/c/2, Radom, Poland), freeze-dryer (Mill rock technology, model BT85, Danfoss, Shanghai, China), filter paper (Machrery-Nagel, Bethlehem, PA, USA; MN 617 and Whatman no.1), and rotary evaporator (Heidolph OB2000, VV2000, Schwabach, Germany).

### 2.2. Chemicals

From Sigma-Aldrich (Schnelldorf, Germany) the following were purchased: dimethyl sulfoxide, p-nitrophenyl butyrate, Orlistat, and tris-HCl buffer; while from Sigma (St. Louis, MO, USA) we purchased porcine pancreatic lipase type II (100–500 units/mg protein (using olive oil (30 min incubation))) and 30–90 units/mg protein (using triacetin)); from Lobachemie (Mumbai, India). We purchased ethanol, acetone, hexane and acetonitrile from SPF (Gurugram, India).

### 2.3. Preparation of Plants Extracts

The required parts from *A. palaestinum*, *C. azarolus*, *M. parviflora*, *T. syriacum*, *R. coriaria*, *R. officinalis*, *P. guajava*, *O. dayi*, *B. nigra*, and *V. vinifera* were collected in May 2016 from different regions of Palestine during the flowering time, except *C. azarolus* fruits which were gathered during the fruiting period of the plant. Botanical identification was carried out at the Pharmacognosy and Herbal Products Laboratory at An-Najah National University, and three samples of each plant were taken for the identification process as well as the voucher specimen codes, including: Pharm-PCT-246, Pharm-PCT-712, Pharm-PCT-1506, Pharm-PCT-2396, Pharm-PCT-2037, Pharm-PCT-2732, Pharm-PCT-2720, Pharm-PCT-1727, Pharm-PCT-408, and Pharm-PCT-2665, respectively. The required parts used from the 10 plants were washed and then dried in the shade at a controlled temperature (25 ± 2 °C) and humidity (55 ± 5 RH). It took about two weeks until all the plant parts became well dried. After drying, the plant materials were well ground into a fine powder by using a mechanical blender and transferred into airtight containers with proper labeling for future use.

### 2.4. Preparation of Plant Extracts for Pancreatic Lipase Inhibition Assay

A total of 25 g of the powdered plant was weighed and then exhaustively extracted by adding 100 mL of n-hexane and 150 mL of 50% ethanol into triply-distilledwater. The mixture was then shaken for 48 h at room temperature using a shaker that was set at 200 rpm. Afterwards, the mixture was filtered using a suction flask and Buchner funnel filtration. The obtained filtrate was separated individually by a separatory funnel into 2 phases—a lower aqueous phase representing the first aqueous extract and an upper organic phase representing the organic extract. The aqueous extract was dried using a freeze-dryer for 48 h. Meanwhile, the organic extracts were placed in a hood at 25 °C to evaporate leftover organic solvents until completely dried. The crude organic and aqueous extracts were stored at 4 °C for further use [1].

### 2.5. Pancreatic Lipase Inhibition

The porcine pancreatic lipase inhibitory assay was adapted from Zheng et al., 2010, and Bustanji et al., (2010) [2,3], with some modifications. 1 mg/mL (1000 μg/mL) plant extract stock solution in 10% DMSO was used, from which five different solutions were prepared with the following concentrations: 50, 100, 200, 300, and 400 μg/mL. 1 mg/mL stock solution of pancreatic lipase enzyme was prepared immediately before being used. This procedure was carried for the ten studied plants species. A stock solution of PNPB (p-nitrophenyl butyrate) was prepared by dissolving 20.9 mg of PNPB in 2 mL of acetonitrile. 0.1 mL of porcine pancreatic lipase (1 mg/mL) was added to test tubes containing 0.2 mL of the various concentrations (50, 100, 200, 300, 400 μg/mL) of plant extract. The resulting mixtures were then made up to 1 mL by adding Tri-HCl solution (pH 7.4) and incubated at 25 °C for 15 min. After the incubation period, 0.1 mL of PNPB solution was then added to each test tube. The mixture was again incubated for 30 min at 37 °C. Pancreatic lipase activity was determined by measuring the hydrolysis of p-nitrophenyl butyrate to p-nitrophenol at 405 nm using a UV-visible spectrophotometer. The same procedure was repeated for the aqueous and organic extracts and for Orlistat (a positive control) using the same concentrations as mentioned above.The established tests were performed in triplicates.

## 3. Results

Twenty crude aqueous and organic extracts were prepared from ten plant species found in the West Bank area of Palestine and their anti-lipase activity was investigated at a concentration of 1000 μg/mL for porcine pancreatic lipase inhibition. The inhibitory activities towards pancreatic lipase are reported in Table 1.

**Figure 1:**
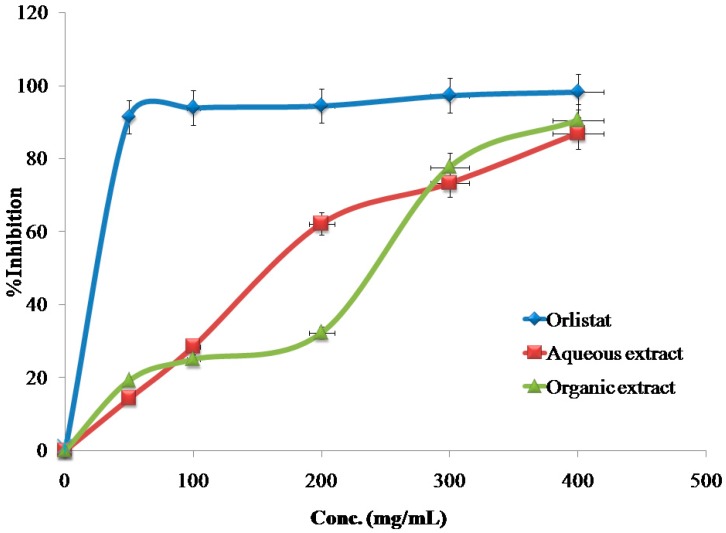
The inhibitory effects of the aqueous and organic extracts of *A. palaestinum* and Orlistat on the activity of porcine pancreatic lipase.

**Figure 2:**
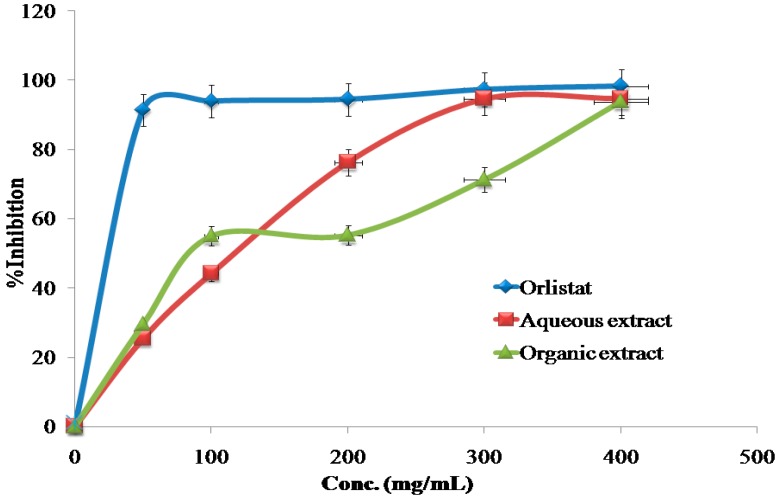
The inhibitory effects of the aqueous and organic extracts of *B. nigra* and Orlistat on the activity of porcine pancreatic lipase.

**Figure 3:**
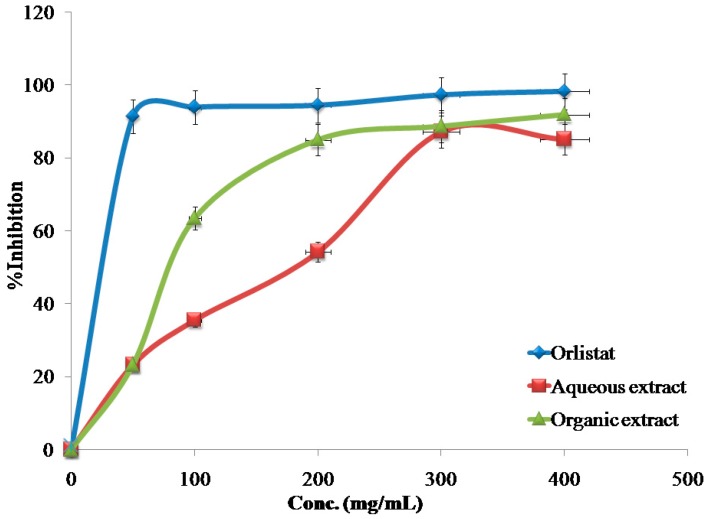
The inhibitory effects of the aqueous and organic extracts of *C. azarolus* and Orlistat on the activity of porcine pancreatic lipase.

**Figure 4:**
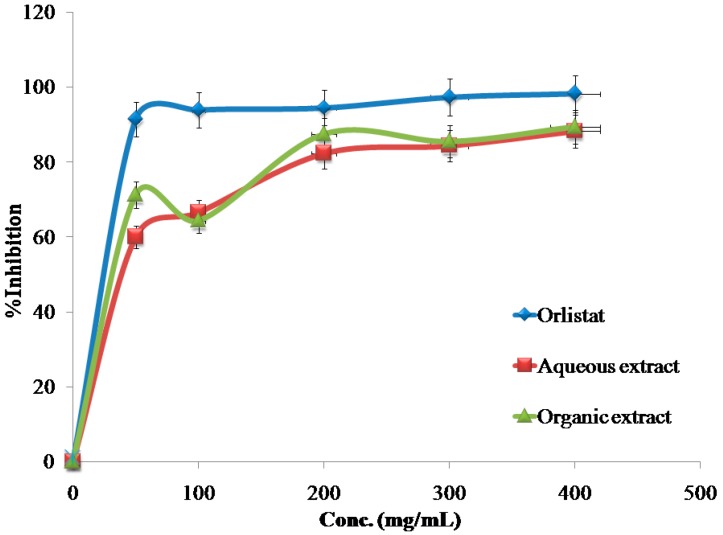
The inhibitory effects of the aqueous and organic extracts of *M. parviflora* and Orlistat on the activity of porcine pancreatic lipase.

**Figure 5:**
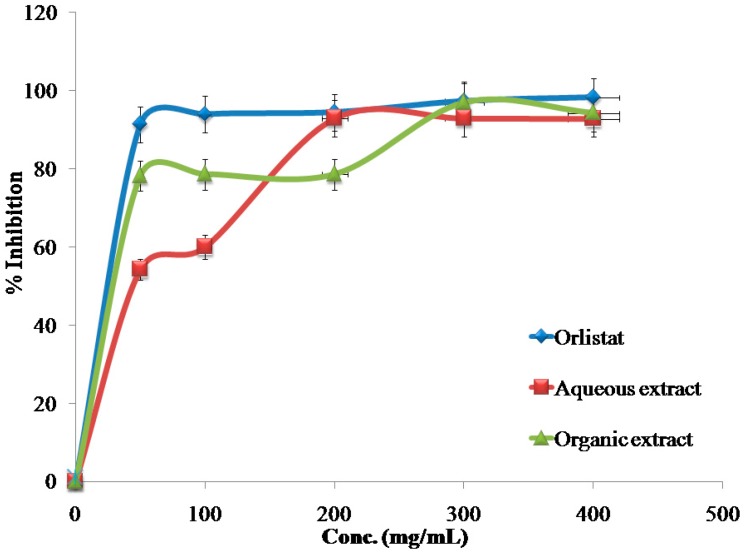
The inhibitory effects of the aqueous and organic extracts of *O. dayi* and Orlistat on the activity of porcine pancreatic lipase.

**Figure 6:**
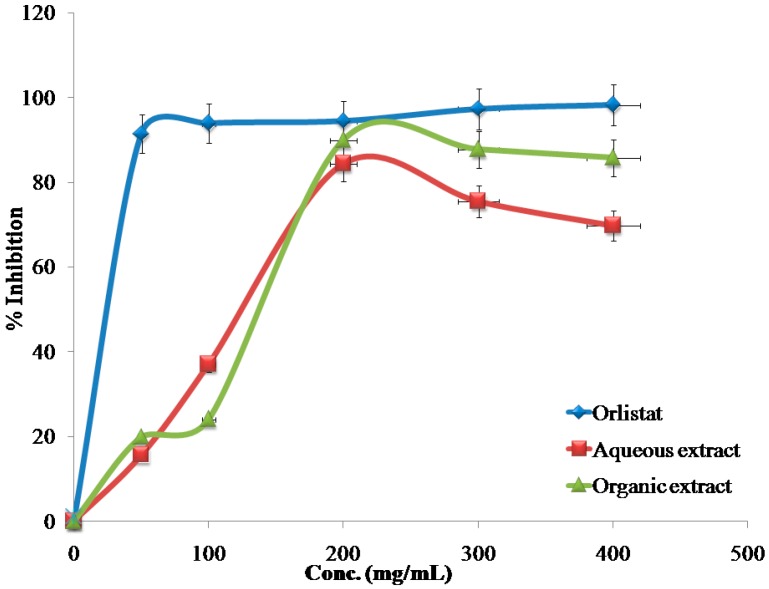
The inhibitory effects of the aqueous and organic extracts of *P. guajava* and Orlistat on the activity of porcine pancreatic lipase.

**Figure 7:**
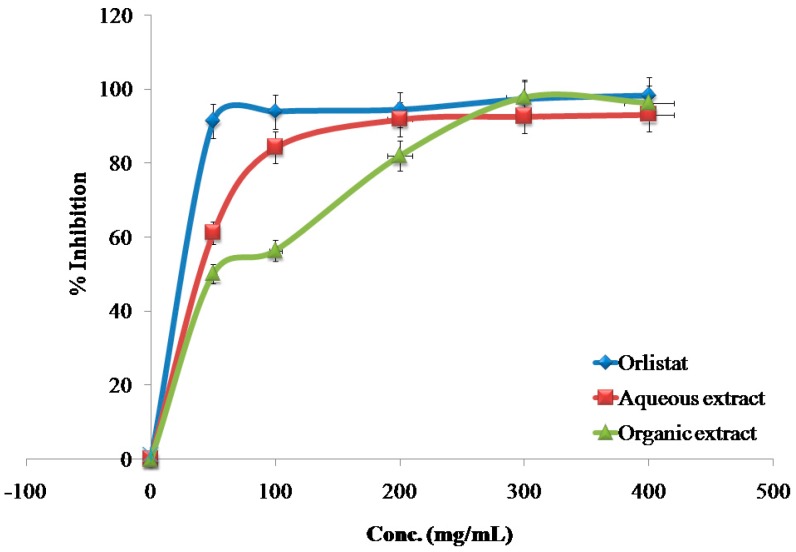
The inhibitory effects of the aqueous and organic extracts of *R. coriaria* and Orlistat on the activity of porcine pancreatic lipase.

**Figure 8:**
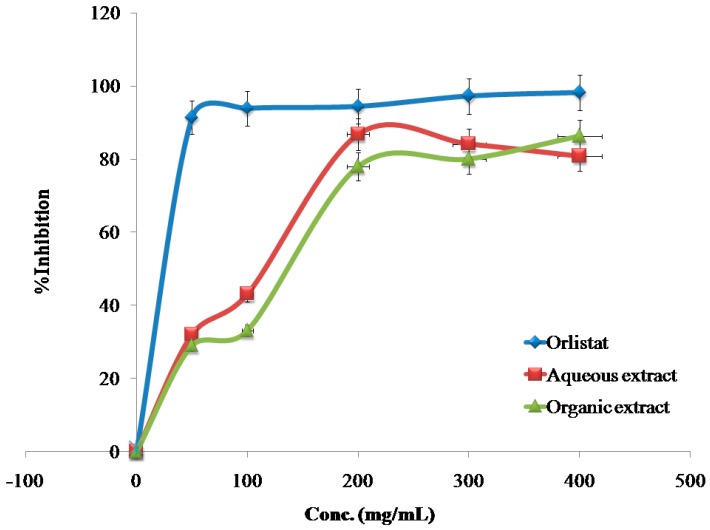
The inhibitory effects of the aqueous and organic extracts of *R. officinalis* and Orlistat on the activity of porcine pancreatic lipase.

**Figure 9:**
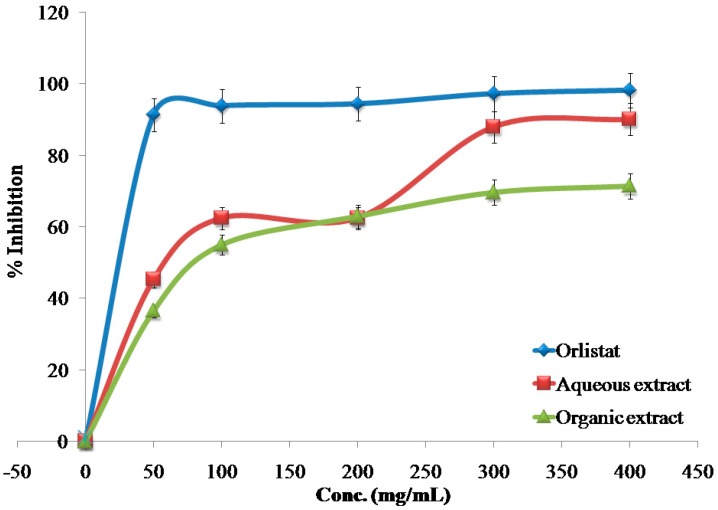
The inhibitory effects of the aqueous and organic extracts of *T. syriacum* and Orlistat on the activity of porcine pancreatic lipase.

**Figure 10:**
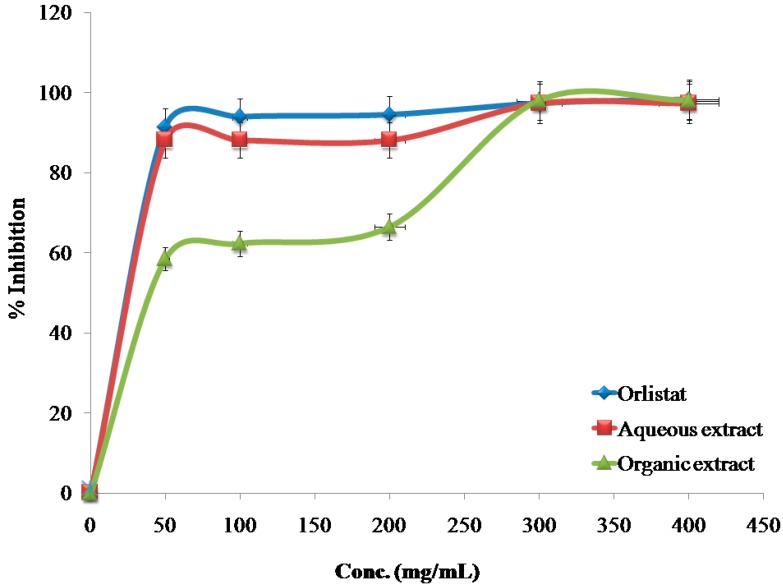
The inhibitory effects of the aqueous and organic extracts of *V. vinifera* and Orlistat on the activity of porcine pancreatic lipase.

The inhibitory effects of the reference drug (Orlistat) and plant extracts aredose-dependent. Different solutions of the Orlistat and plant extracts were prepared in escalating doses as shown in Table 1. The activity of lipase decreased by increasing the concentration of Orlistat and the plant extracts. TheIC_50_ values for the drug and plant extracts were calculated and the degree of lipase inhibition was plotted as shown in Figure 1, Figure 2, Figure 3, Figure 4, Figure 5, Figure 6, Figure 7, Figure 8, Figure 9 and Figure 10. The IC_50_ values represent the concentration of the inhibitors at which 50% of the enzyme is inhibited and it is generally used to express the inhibitory effect of the lipase enzyme.

Among the 20 plant extractsexamined, seven crude extracts at a concentration 100 μg/mL significantly inhibited porcine pancreatic lipase activity in vitro as demonstrated using a p-nitrophenyl butyrate-based assay. Throughout the investigated results, the aqueous extracts of *V. vinifera* and *R. coriaria,* with IC_50_ values of 14.13 and 19.95 μg/mL, respectively, showed the highest porcine pancreatic lipase inhibitory effects of all the studied aqueous extracts. Meanwhile, the organic extract of *O. dayi*, with an IC_50_ value of 18.62 μg/mL, showed the highest porcine pancreatic lipase inhibitory effects of all studied organic extracts.

In addition to that, the studied extracts’ IC_50_ values were compared with the standard antilipase compound Orlistat, which has an IC_50_ value of 12.38 ± 2.3 μg/mL. These results, and the results of all of the 10 studied plants, are well clarified in Table 1 and in Figure 1, Figure 2, Figure 3, Figure 4, Figure 5, Figure 6, Figure 7, Figure 8, Figure 9 and Figure 10.

## 4. Discussion

In this study, ten traditional edible medicinal plants, including *T. syriacum, O. dayi, M. parviflora, B. nigra, V. vinifera, C. azarolus, R. coriaria, A. palaestinum, P. guajava*, and *R. officinalis*, were assessed for their activity as porcine pancreatic lipase inhibitors. In many countries such as Palestine, Jordan, Iraq, Greece, and Pakistan, most of these plants were reported as being used as traditional medicines for the treatment of obesity [4,5,6,7,8,9,10,11,12,13,14,15,16].

Obesity is a chronic metabolic disorder caused by an imbalance between energy intake and expenditure. It is a major risk factor for cancer as well as endocrine, metabolic, and cardiovascular disorders [17,18,19]. Accordingly, the use of functional foods, such as the consumption of edible plants, would be a great and safe medicinal alternative in the treatment of obesity. They have been targeted to promote beneficial health effects, especially for the prevention of pathophysiological conditions such as obesity, dyslipidemia, diabetes, hypertension, and cancer [20,21].

The lipolytic pancreatic lipase enzyme is synthesized and secreted by the pancreas, plays a key role in the efficient digestion of lipids, and is responsible for the hydrolysis of 50–70% of total dietary lipids. The anti-lipase effect is one of the most widely studied mechanisms in determining the potential efficacy of natural products as anti-obesity agents [22,23].

Recently, global attention has been focused on the effects of plants, especially those that classified as traditional medicinal and edible types for the treatment of obesity and for controlling of overweight due to their safety issue, as well as these plants have been consumed from ancient times and their toxic and other side effects have been observed and documented if present [24].

In addition to this, Palestinian territory is covered with a huge number of plants and from this huge biodiversity, there are large numbers of them considered traditional medicines or foodssinceancient times [25]. From these plants, *A. palaestinum*, *C. azarolus*, *M. parviflora*, *T. syriacum*, *R. coriaria*, *R. officinalis*, *P. guajava*, *O. dayi*, *B. nigra*, and *V. vinifera* were investigated for their efficacy as natural anti-obesity products and compared with Orlistat, which considered the anti-lipase drug of choice [26].

Orlistatis a potent inhibitor of pancreatic lipase enzyme isolated from bacteria *Streptomyces toxytricini*. This prescription drug is designed to treat obesity by lowering lipid digestion and is produced under the trade name Xenical^®^ by Roche [27].

Our obtained results showed that the IC_50_ values of the aqueous extracts of *V. vinifera* and *R. coriaria* have high porcine pancreatic lipase inhibition potential with IC_50_ values of 14.13 and 19.95 μg/mL, respectively. The IC_50_ value of the organic extract of *O. dayi* was 18.62 μg/mL, in comparison with the reference compound (positive control) Orlistat which has an IC_50_ of 12.38 μg/mL.

Moreover, the *R. coriaria* (Sumac) fruit’s aqueous extract showed potential antilipase effects with an IC_50_ value 19.95 μg/mL, and this plant is an edible one and grows wildly in tropical and temperate regions worldwide, often growing in agriculturally-in viable regions. *R. coriaria* belongs to the Anacardiaceae family and is used in Turkey as a folk medicine for the treatment of diabetes due to its ability to cure various diabetes complications [28].

A study performed by Golzadeh et al., (2012), showed that Sumac reduced total cholesterol, triglyceride, low-density lipoprotein, and blood sugar levels in animal studies [29].

Accordingly, these results were confirmed in our in-vitro study which confirmed its activity as an anti-lipase agent.

In addition to these results, the organic extract of *O. dayi* also showed potential anti-lipase effects with an IC_50_ value 18.62 μg/mL. It is a perennial, endemic, culinary plant, and is considered a desert plant that is mainly distributed in African and Mediterranean regions [30,31]. It is used as a tea, a spice, and boiled with meat also used in salads in many countries, reflecting its therapeutic effect of reducing lipid absorption in the gastrointestinal tract [32,33].

*Vitis vinifera* belongs to the Vitaceae family, whose origin is Mediterranean, and is considered one of most common plant species cultivated worldwide and one of the most important economical plants because it is used to produce wine, table grape juice, and raisins [34,35].

The leaves of *V. vinifera* consumed with rice and meat and considered one of the most popular culinary dishes in Arabian countries, as well as other countries such as Turkey and Italy [36,37,38]. This plant’s leaves showed a powerful anti-lipase effect in comparison with Orlistat and have an IC_50_ value 14.13 μg/mL.

In a study which was conducted on the aqueous extract of *V. vinifera* leaves by Fernandes et al., (2013), it was found that the plant’s leaves are rich in phenolic compounds, including phenolic acids and flavonoids such as trans-caffeoyl tartaric acid, trans-coumaroyl tartaric acids, myricetin-3-*O*-glucoside, quercetin-3-*O*-glucoside, quercetin, kaempferol-3-*O*-glucoside, quercetin-3-*O*-galactoside, kaempferol, and isorhamnetin [34].

In addition, *R. coriaria* fruits have high concentrations of phenolic compounds, including caffeoylquinic acid, quercetin, rhamnetin, myricetin, kaempferol, gallic acid, ellagic acid, methyl gallate, m-digallic acid, amenthoflavone, agathisflavone, hinokiflavone, and sumaflavone [39,40].

*Origanum dayi* is an endemic subshrub plant species that grows in the West Bank area of Palestine. The leaves of *O. dayi* contain a mixture of volatile oils such as terpinen-4-ol, α-terpineol, 1,8-cineole, (E)-sabinene hydrate, (E)-sabinene hydrate acetate, and linalyl acetate [30,31]. To the best of the authors’ knowledge, there were no previous studies in the literature about the chemical constituents of *O. dayi*, except on the volatile oils. However, many phenolic compounds were isolated from other species of *Origanum* (*O. vulgare*), including protocatechuic acid, caffeic acid, 2-caffeoyloxy-3-[2-(4-hydroxybenzyl)-4,5-dihydroxy]phenylpropionic acid, and phenyl glucoside [41].

In fact, in many previous studies, it was reported that flavonoids and other phenolic compounds act as porcine pancreatic lipase enzyme inhibitors by binding to the enzyme−substrate complex, reducing the lipid absorption [42,43].

To the best of the authors’ knowledge, there were no previous studies on the effects of the aqueous and organic extracts of *V. vinifera*, *R. coriaria* and *O. dayi* on the lipase enzyme and our study is the first one on these plants. Further pharmacological in-vivo studies are required to confirm these findings and to identify the key chemical elements in these plants, using chromatographical isolation of the bioactive molecules, responsible for these pharmacological effects.

## 5. Conclusions

Obesity has reached epidemic proportions and is becoming a public health concern of the highest order. Several methods are now available to treat obesity and Orlistat is the most used one. Several plants are used in folk medicine to treat obesity and the use of edible plants is of great importance since they have few of the adverse effects that may be encountered with chemical or drug treatments. Our obtained results showed that some edible plants could replace Orlistat in the treatment of obesity. According to the obtained results, *V. vinifera, R. coriaria,* and *O. dayi* can be used as natural inhibitors of pancreatic lipase and so are new players in obesity treatment. In fact, these plants can be freely and safely consumed in the daily diet, be prepared as natural supplements to treat or prevent obesity and control weight gain, and can be used for the treatment of hyperlipidemia.

## Figures and Tables

**Table 1:** Porcine pancreatic lipase inhibitory properties, expressed as IC_50_ (μg/mL), and yield percentages of the aqueous and organic extracts of 10 plant species that were collected from different regions in Palestine.

Aqueous Extract Yield,%	IC_50_ of the Aqueous Extract, μg/mL	Organic Extract Yield,%	IC_50_ of the Organic Extract, μg/mL	Parts Used	Family	Local Name	Studied Plants’
							Latin Names
18.8	107.2 ± 2	2.7	147.9 ± 2	Leaves	Araceae	Loof	*A. palaestinum*
9.8	83.2 ± 1.9	1.8	40.7 ± 1.8	Leaves	Rosaceae	Zaaror	*C. azarolus*
6.2	28.2 ± 2.4	0.6	23.7 ± 3	Leaves	Malvaceae	Khobeze Baladi	*M. parviflora*
18.6	39.8 ± 1.8	0.7	74.1 ± 2.2	Leaves	Compositae	Ejr Alasad	*T. syriacum*
14.4	19.95 ± 2.8	2.8	30.2 ± 1.5	Fruits	Anacardiaceae	Sumak	*R. coriaria*
7.2	51.3 ± 2.4	7.2	65 ± 2	Leaves	Lamiaceae	Hasaa Alban	*R. officinalis*
9.8	87.1 ± 1.4	9	64.6 ± 2	Leaves	Myrtaceae	Jawafa	*P. guajava*
19.9	26.9 ± 2	1.5	18.6 ± 2.6	Leaves	Lamiaceae	Albardaqosh	*O. dayi*
26	47.9 ± 2.4	1.4	66.1 ± 2.1	Leaves	Brassicaceae	Khardal	*B. nigra*
12.5	14.1 ± 1.9	0.6	28.8 ± 2.5	Leaves	*Vitaceae*	Anab	*V. vinifera*
